# 2-Methoxyestradiol ameliorates doxorubicin-induced cardiotoxicity by regulating the expression of GLUT4 and CPT-1B in female rats

**DOI:** 10.1007/s00210-024-03073-z

**Published:** 2024-04-23

**Authors:** Mohamed H. Sobhy, Ahmed Ismail, Mohammed S. Abdel-Hamid, Mohamed Wagih, Marwa Kamel

**Affiliations:** 1 Department of Pharmacology and Toxicology, Faculty of Pharmacy, Al-Azhar University, Cairo, Egypt; 2 Nanomedicine Research Labs, Center for Materials Science, Zewail City of Science and Technology, 6th of October City, Giza, Egypt; 3 Department of Biochemistry and Molecular Biology, Faculty of Pharmacy, Al-Azhar University, Cairo, Egypt; 4 Department of Pharmacology and Toxicology, Faculty of Pharmacy, Fayoum University, Fayoum, Egypt; 5 Department of Pathology, Faculty of Medicine, Beni-Suef University, Beni-Suef, Egypt; 6 Department of Cancer Biology, Unit of Pharmacology and Experimental Therapeutics, National Cancer Institute, Cairo University, Cairo, Egypt

**Keywords:** Doxorubicin, Cardiotoxicity, Estrogen, 2-Methoxyestradiol, GLUT4, CPT-1B

## Abstract

The clinical usage of doxorubicin (DOX) is hampered due to cardiomyopathy. Studies reveal that estrogen (E2) modulates DOX-induced cardiotoxicity. Yet, the exact mechanism is unclear. The objective of the current study is to evaluate the influence of E2 and more specifically its metabolite 2-methoxyestradiol (2ME) on cardiac remodeling and the reprogramming of cardiac metabolism in rats subjected to DOX cardiotoxicity. Seventy-two female rats were divided into groups. Cardiotoxicity was induced by administering DOX (2.5 mg/kg three times weekly for 2 weeks). In some groups, the effect of endogenous E2 was abolished by ovariectomy (OVX) or by using the estrogen receptor (ER) blocker Fulvestrant (FULV). The effect of administering exogenous E2 or 2ME in the OVX group was studied. Furthermore, the influence of entacapone (COMT inhibitor) on induced cardiotoxicity was investigated. The evaluated cardiac parameters included ECG, histopathology, cardiac-related enzymes (creatine kinase isoenzyme-MB (CK-MB) and lactate dehydrogenase (LDH)), and lipid profile markers (total cholesterol (TC), triglyceride (TG), and high-density lipoprotein (HDL)). The expression levels of key metabolic enzymes (glucose transporter-4 (GLUT4) and carnitine palmitoyltransferase-1B (CPT-1B)) were assessed. Our results displayed that co-treatment of E2 and/or 2ME with DOX significantly reduced DOX-induced cardiomyopathy and enhanced the metabolism of the heart through the maintenance of GLUT4 and CPT-1B enzymes. On the other hand, co-treatment of DOX with OVX, entacapone, or FULV increased the toxic effect of DOX by further reducing these important metabolic enzymes. E2 and 2ME abrogate DOX-induced cardiomyopathy partly through modulation of GLUT 4 and CPT-1B enzymes.

**Graphical Abstract:**

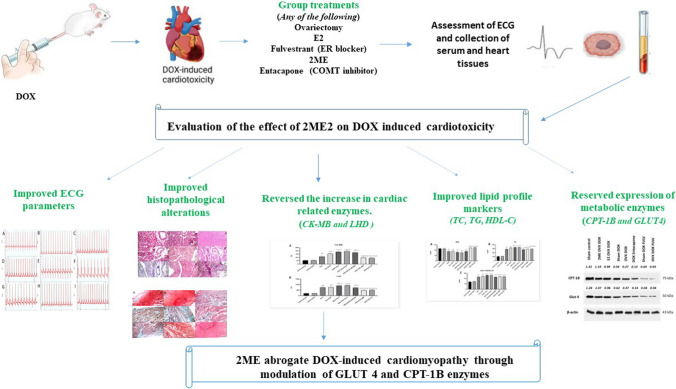

**Supplementary Information:**

The online version contains supplementary material available at 10.1007/s00210-024-03073-z.

## Introduction

Doxorubicin (DOX) is an effective chemotherapeutic agent; however, it can cause cardiotoxicity which limits its clinical use (Octavia et al 2012). This cardiotoxicity is dose dependent and cumulative with different pathways included, and despite continuous research, the mechanisms clarifying DOX cardiotoxicity are not completely understood (Rawat et al 2021).

A potential pathway is that DOX causes a shift in metabolic reprogramming of fatty acid and glucose metabolism which is imparted in cardiotoxicity (Russo et al. 2021). The heart requires constant energy and metabolic substrates. Fatty acid is the major metabolic substrate for the normal adult heart, but the heart changes preference from fatty acid to glucose in several conditions (Shao and Tia 2015), where it was found that cardiac metabolic modifications of fatty acids, glucose, and lactate are early signs of cardiac stress (Zhang et al 2013). Glucose transporter (GLUT) family proteins are important glucose transporters where GLUT4 is a major transporter in newborns and adult hearts (Joost and Thorens 2001). It is found mostly in intracellular membranes. In the presence of stimuli, such as insulin, catecholamines, and ischemia, GLUT4 is translocated to the cell surface; thereby, transportation of glucose into the cardiomyocytes rises (Szablewski 2017). On the other hand, carnitine palmitoyltransferase (CPT) catalyzes the transference of long- and medium-chain fatty acids from cytoplasm into mitochondria where they are oxidized. Deficiency of CPT enzyme causes diseases of fatty acid metabolism. There are two subforms of CPT: CPT-1 at the external membrane of the mitochondria and CPT-2 inside the mitochondria (Joshi and Zierz 2020). CPT-1B is the muscle isoform predominantly existing in the heart.

Studies reveal that women maintain better cardiac function than men do (Meiners et al 2018; Wilcox et al 2022) and that female sex hormones protect against DOX cardiotoxicity (Moulin et al 2015). For example, estrous-staged treatment decreases cardiotoxicity produced by DOX in hypertensive female rats, and exogenously administered estrogen (E2) could suppress DOX toxicity in ovariectomized hypertensive female rats (Pokrzywinski et al 2018), or even in male rats (Zhang et al 2017). However, the reason behind the sexual dimorphism in DOX cardiotoxicity needs further investigation.

2-Methoxyestradiol (2ME) is an active metabolite of E2 generated endogenously through several conversion reactions involving catechol-o-methyl transferase enzyme (COMT) and is present in both males and females. Additionally, it is available as a drug candidate possessing potential antitumor effects against a wide range of tumors (Amye et al 2009; Daniela et al 2009; Mengyu and Yongtao 2020). Most importantly, it does not display any estrogenic activity due to its weak affinity to estrogen receptors, which helps to evade estrogen-dependent diseases such as breast cancer (LaVallee et al 2002). Interestingly, 2ME resensitizes DOX-resistant breast cancer cells (Mueck and Seeger 2010; Yu et al 2022a, b) with possible cardiovascular benefits especially on lipid profile (Bourghardt et al 2007; Rios and Touyz 2021) and the maintenance of glucose homeostasis in mice (Kanasaki et al 2017). Consequently, 2ME has entered scientists’ field of vision.

Thus, our aim is to demonstrate the influence of E2 and, most specifically, 2ME as protective agents against DOX-induced cardiomyopathy and remodeling and the involvement of metabolic reprogramming in this effect.

## Materials and methods

### Animals

Twelve-week-old female Sprague-Dawley rats (200 ± 20 g) were obtained from El-Nile Co. for Pharmaceutical and Chemical Industries (Cairo, Egypt). Fourteen days before performing the experiments, animals were allowed to adapt to facility conditions at 25±2 °C, 50–70% humidity, and 14-/10-h light and dark cycles. Normal chow diet and drinking water were supplied ad libitum. The study was approved by the Institutional Animal Care and Use Committee, Cairo University (CU-IACUC), with an approval date of May 2023 and approval number CU III F 24 23.

### Drugs and chemicals

The following chemicals were obtained: DOX (Hikma Pharmaceuticals, Egypt); COMT inhibitor Entacapone (Orion Pharma, Espoo, Finland); E2 (Sigma-Aldrich Co, St. Louis, MO, USA); 2ME (Fraken Biochem Co. Ltd., Qingdao, China); E2 blocker Fulvestrant (FULV) (Astra Zenica, UK); CPT-1B antibody (22170-1-AP); and GLUT4 antibody (21048-1-AP) (Protein tech, Manchester, UK). Additional used chemicals were of analytical grades.

### Ovariectomy surgery

Female rats were ovariectomized as formerly described (Khajuria et al. 2012). In brief, female rats underwent anesthesia using thiopental (25 mg/kg, i.v), and then, the surgery region was cleaned and shaved. Above the urinary outlet in the abdominal middle line, a slit was done. The ovary and uterine horn were identified. Then, a braided silk suture was made around the distal uterine horns (Ethicon mersilk sutures 3/0). This was sectioned afterwards, and the ovaries were detached. Finally, the skin was sutured.

### Experimental design and treatment protocol

Several groups were designed to test our hypothesis. DOX was used to induce and set the cardiotoxicity model in rats. For comparisons, the effect of estrogen was abolished by both surgery (OVX) and chemically using the estrogen receptor (ER) blocker FULV. FULV was also used to study whether E2 protects against cardiotoxicity by non-ligand-dependent mechanisms. In other groups, exogenous E2 was administered, and its effect was evaluated. Moreover, the effect of 2ME treatment was assessed. Endogenously, 2ME is mainly formed due to the activity of the COMT enzyme. Therefore, the 2ME effect was tested by administering the COMT inhibitor Entacapone in one group, as well as by administering exogenous 2ME in another group. The 2ME groups would also give an indication of whether the effect of E2 is mediated through its metabolite 2ME. Thus, 72 female rats were allocated randomly as shown:

- Group 1: Control sham group, undertook surgical technique without OVX
- Group 2: Control OVX group, underwent OVX surgical procedure without any other treatment
- Group 3: Sham+ DOX group
- Group 4: OVX +DOX group
- Group 5: Sham+ DOX+ FULV group
- Group 6: OVX+ DOX+ FULV group
- Group 7: Sham+ DOX + entacapone group
- Group 8: OVX+ DOX+ 2ME group
- Group 9: OVX+ DOX+ E2 group

The experiment lasted for 2 weeks provided that OVX surgery began 6 weeks before the start of the experiment. Also, the first dose of FULV was 1 week before the experiment. Doses used were as follows: DOX, 2.5 mg/kg i.v three times weekly for 2 weeks (Babaei et al 2020); FULV, 10 mg/kg IM once weekly for 3 weeks in separate days from DOX treatment (Yamamoto et al 2019); entacapone, 200 mg/kg orally once daily for 2 weeks (Yuan et al 2013); 2ME, 20 mg/kg i.p. once daily for 2 weeks (Chen et al 2014); E2, 30 µg/kg s.c once daily for 2 weeks (Abd El-Lateef et al 2019). The administration of entacapone, 2ME, and E2 was in the morning, while that of DOX was at night after them. Regarding the administration of FULV, it was performed at the beginning of every week, on separate days from DOX treatment. An average of eight animals were assigned to each group (*n*=8).

Twenty-four hours after the last dose of DOX, body weights were documented, and electrocardiogram (ECG) data were recorded under anesthesia using thiopental (i.v 5 mg/kg). Blood samples were taken from retroorbital plexus and sera were collected by centrifugation at 1500×g for 10 min at 4 °C which were then utilized for biochemical analysis. Subsequently, rats were euthanized by the cervical dislocation method, and the heart tissues were dissected and weighed. Sections of the heart were conserved in a 10% buffered formalin solution and sent for histopathological evaluation. Another section was stored at −80 °C for western blotting.

### Assessed parameters

#### Assessment of ECG

The rats were anesthetized using thiopental (25 mg/kg i.v) 24 h after the final dose of DOX and then positioned on the ECG using three leads with limbs taped to the leads. Uninterrupted ECG recording was obtained and checked automatically using the ECG Analyze software lab chart provided by AD instruments.

#### Assessment of cardiac-related enzymes

Serum level activities of creatine kinase isoenzyme-MB (CK-MB) and lactate dehydrogenase (LDH) were evaluated by using kits from Spinreact (St. Esteve d’en Bas, Girona, Spain).

#### Histopathological examination

Heart tissues from different groups were fixed in a 10% buffered formalin solution for 1 week, dehydrated in ascending grades of ethanol, cleared in xylene, and then embedded in paraffin. Sections were obtained from each block at a thickness of 4 µm. One section was stained with hematoxylin and eosin (H&E) and the other with Masson’s trichrome (MTC). Sections were studied using Nikon Lapophot Light. Images were taken and processed using Adobe Photoshop version 8.0.

For H&E, the score was as follows: 1 = low, 2 = moderate, 3 = high, and 4 = severe.

MTC staining was graded according to the following: 1, 2 = heart showed average collagen distribution in the myocardium and around blood vessels; 3, 4 = mild fibroplasia in the myocardium and/or around blood vessels; 5, 6 = moderate fibroplasia in the myocardium and/or around blood vessels; and 7, 8 = severe fibroplasia in the myocardium and/or around blood vessels.

#### Determination of lipid profile markers

The concentrations of total cholesterol, triglyceride (TG), and high-density lipoprotein cholesterol (HDL) were determined enzymatically by colorimetric kits (BIOMED Diagnostic, Cairo, Egypt), following manufacturer instructions.

#### Western blot analysis

Protein expression of CPT-1B and GLUT4 was estimated using western blot analysis. In short, equal amounts of protein were extracted from the heart tissues by ReadyPrep™ kit (Bio-Rad Laboratories, CA, USA). The method depends on the presence of the strongly chaotropic extraction solution containing the zwitterionic detergent ASB-14, providing powerful solubilizing reagents for 2-D electrophoresis. Briefly, 2-D rehydration/sample buffer 1 (7 M urea, 2 M thiourea, 1% (w/v) ASB-14 detergent, 40 mM Tris base, and 0.001% bromophenol blue) and tributylphosphine (TBP) reducing agent (200 mM TBP in 1-methyl-2-pyrrolidinone sealed under nitrogen gas) were prepared according to manufacturer’s instructions. Immediately before performing the extraction, the complete buffer was prepared by adding TBP reducing agent and the ampholyte to the reconstituted 2-D rehydration/sample buffer 1. Then, 1 ml of complete 2-D rehydration/sample buffer was added per 50–100 mg of tissue. After that, the sample was placed on ice and the suspension was sonicated four times until complete lysis, and placed briefly on ice between each ultrasonic treatment. This was followed by centrifugation for 30 min to remove cell debris, and the supernatant was kept for protein measurement by Bradford protein assay kit (Bio basic, Markham Ontario, Canada). Polyacrylamide gels were prepared using TGX Stain-Free™ FastCast™ Acrylamide Kit (Bio-Rad Laboratories Inc). The Bio-Rad Trans-Blot technology was then used to transfer the protein to PVDF membranes. The blocking step was done by placing the membrane for 1 h at room temperature in tris-buffered saline with Tween 20 (TBST) buffer and 3% bovine serum albumin (BSA). This was followed by incubation with primary antibodies for GLUT4 and CPT-1B (Proteintech, IL, USA). Afterwards, the blot was rinsed and reincubated with peroxidase-labeled secondary antibodies for 1 h at 37 °C (Clarity TM Western ECL substrate Bio-Rad, CA, USA) and β-actin was used as an internal control. The chemiluminescent signals were caught by a CCD camera-based imager. Image analysis software was utilized to record the band intensity of the target proteins normalized against β-actin on the ChemiDoc MP imager (Bio-Rad, CA, USA).

### Statistical analysis

Data for ECG, cardiac-related enzymes, and lipid profile were presented as means ± standard deviation of the mean (SD). Multiple comparisons were done by one-way analysis of variance (ANOVA) followed by the Tukey-Kramer post hoc test. Data for histopathological examination were presented as median ± interquartile range and statistically analyzed by Kruskal-Wallis test (one-way nonparametric ANOVA test) followed by Dunn’s post hoc test and adjusted for multiple comparisons with the Benjamini–Hochberg false discovery rate. The GraphPad Prism (ISI® software, USA) version 5 was used for data analysis and graph presentation.

## Results

### E2 and 2ME decreased DOX-induced ECG changes

**Fig. 1:**
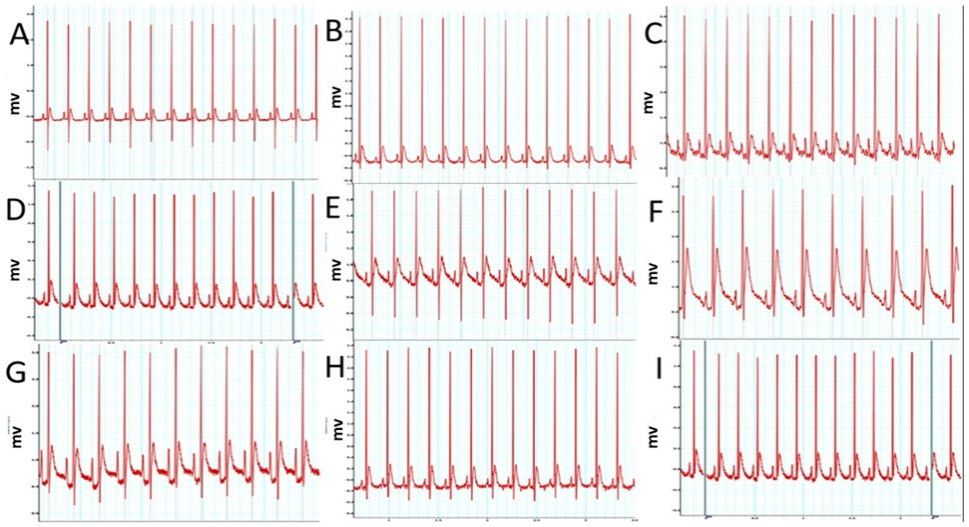

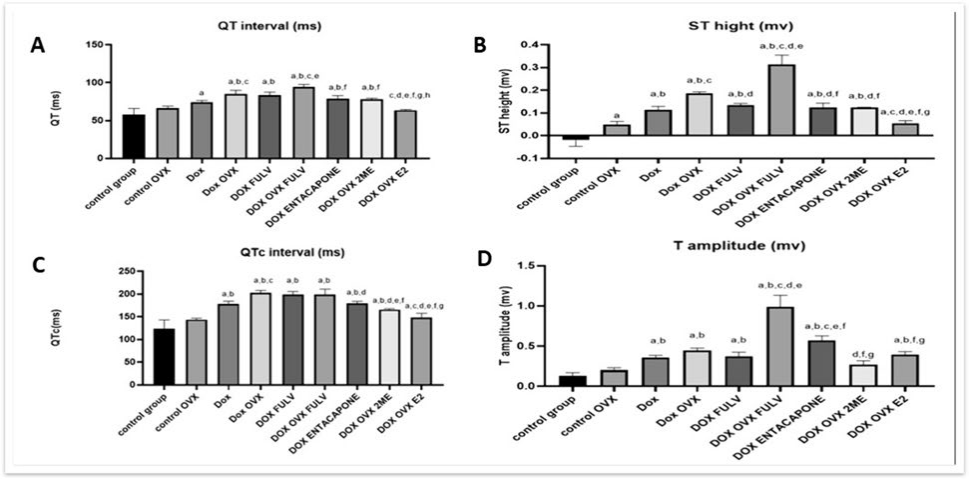
(i) Effect of different treatments on ECG. A, control sham rats; B, control OVX; C, sham + DOX; D, OVX + DOX; E, sham + DOX + FULV; F, OVX + DOX + FULV; G, sham + entacapone; H, OVX + DOX + 2ME; I, OVX + DOX + E2. (ii) Quantitation of the effect of different treatments on relevant ECG parameters. A, QT interval; B, ST heights; C, QTc interval; and D, T amplitude. a, significance from control group A; b, significance from control OVX group B; c, significance from DOX-treated group C; d, significance from DOX + OVX group D; e, significance from DOX + FULV group E; f, significance from DOX + OVX + FULV group F; g, significance from DOX + entacapone group G; h, significance from OVX + DOX + 2ME group H. Data represent mean ± SD. Multiple comparisons were analyzed by ANOVA followed by Tukey-Kramer as a post hoc test (n =8), p ≤ 0.05

To evaluate the influence of E2 on DOX-induced changes in heart rhythms, an ECG was done. Our data in Fig. 1i and ii showed that ovariectomizing female rats did not alter significantly most ECG parameters including QT_c_, QT, T amp, and ST segment. DOX treatment on sham animals caused both QT_c_ and QT prolongation with an increase in T wave amplitude and ST segment elevation; meanwhile, OVX exacerbated DOX toxicity compared to DOX only group with more QT_c_ and QT prolongation with additional ST segment elevation. The ER blocker did not have much change from the DOX OVX group regarding QT_c_ and QT intervals but OVX with ER blocker acutely elevated ST height and T amplitude with QT prolongation. Interestingly, the treatment with DOX in chemically and surgically ovariectomized animals led to a more pronounced alteration in ECG regarding QT, ST, and T amplitude when compared to other groups. The treatment with entacapone significantly elevated T amplitude with no other significant effects compared to the DOX group. In contrast, the treatment with E2 and its metabolite 2ME lowered ST height and QTc duration compared to both OVX DOX and OVX DOX FULV groups, while E2 did not show much change from the OVX DOX group in T amplitude.

### E2 and 2ME reversed the DOX-induced rise in cardiac-related enzymes

**Fig. 2:**
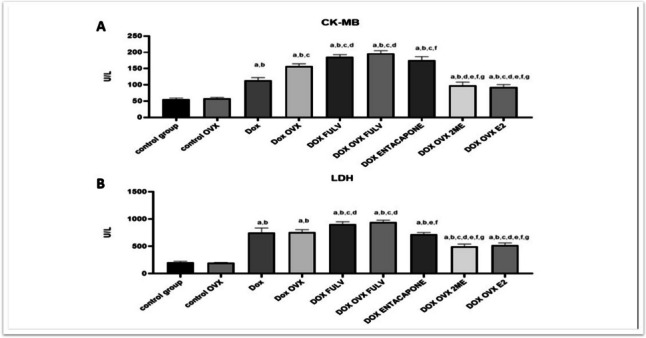
Effect of treatments on cardiac-related enzymes. **A** CK-MB and **B** LDH. a, significance from control group A; b, significance from control OVX group B; c, significance from DOX-treated group **C**; d, significance from DOX + OVX group **D**; e, significance from DOX + FULV group **E**; f, significance from DOX + OVX + FULV group **F**; g, significance from DOX + Entacapone group **G**; h, significance from OVX + DOX + 2ME group **H**. Data represent mean ± SD. Multiple comparisons were analyzed by ANOVA followed by Tukey-Kramer as a post hoc test (n = 8), p ≤ 0.05

To illustrate the consequences of DOX-induced ECG changes, the cardiac enzymes, CK-MB, and LDH levels were determined. Our results illustrated in Fig. 2A and B showed that OVX of female rats without DOX did not significantly alter the levels of both CK-MB and LDH. DOX alone on sham animals increased serum levels of both enzymes. Besides, DOX treatment in chemically and/or surgically ovariectomized animals significantly increased the serum level of CK-MB and LDH when compared to DOX group. Entacapone increased serum CK-MB compared to DOX group with no effect on LDH. In contrast, E2 and its metabolite 2ME declined serum levels of both CK-MB and LDH with no clear difference between them.

### E2 and 2ME alleviated histopathological alterations induced by DOX on cardiac tissues

**Fig. 3:**
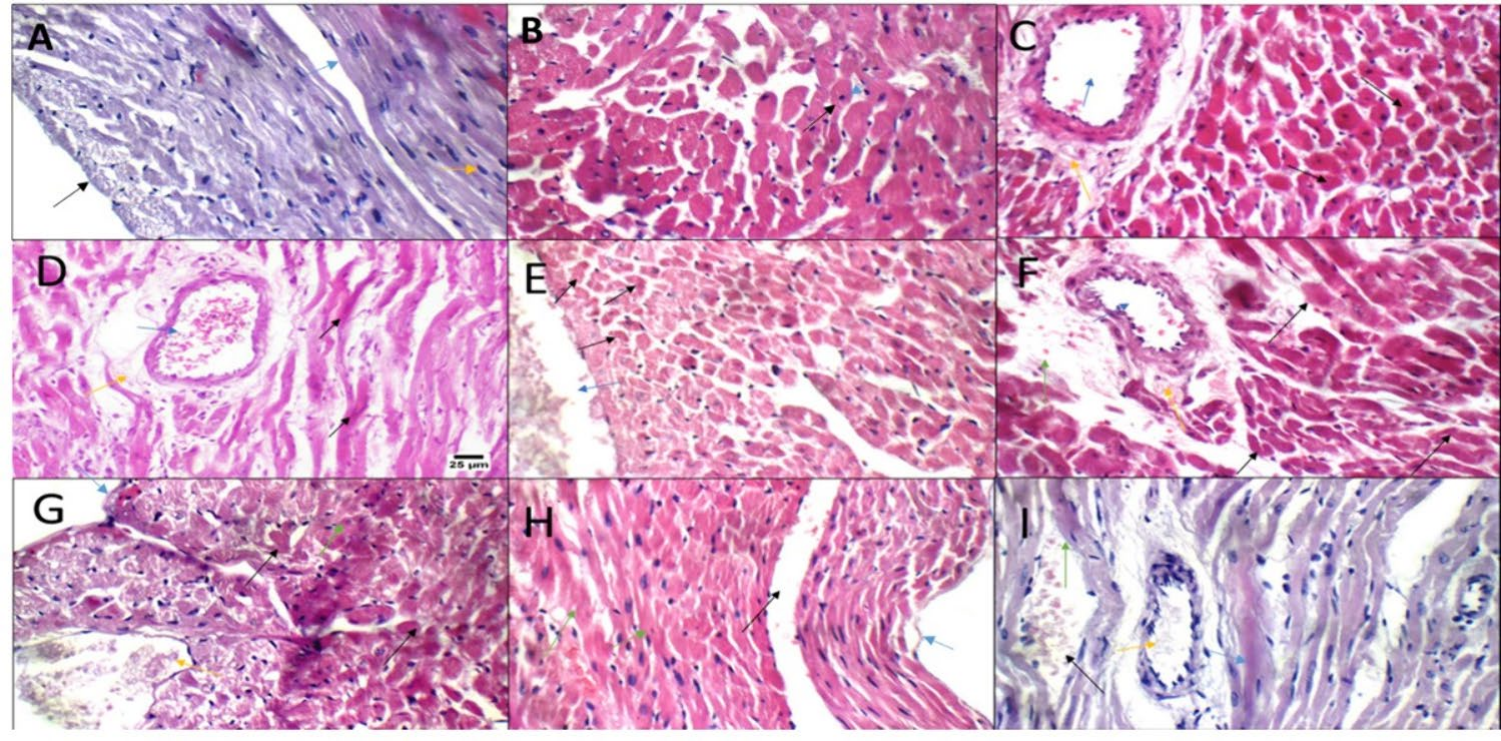
H&E staining of cardiac tissues following different treatments. **A** Control sham rats showing normal cardiac tissue with unremarkable change (black arrow, intact pericardium; blue arrow, viable cardiac muscle fibers with distinct cell borders; yellow arrow, central oval\elongated nuclei); **B** control OVX rat demonstrating normal cardiac tissue with no distinct change (black arrow, distinct cell wall; blue arrow, oval centered nucleus); **C** sham + DOX (blue arrow, moderate dilated blood vessel; yellow arrow, perivascular edema; black arrow, apoptosis); **D** OVX + DOX (blue arrow, moderate dilated blood vessel; yellow arrow, perivascular edema; black arrows, severe apoptosis); **E** sham + DOX + FULV (black arrow, apoptotic cardiac muscle fibers; blue arrow, markedly dilated congested blood vessel); **F** OVX + DOX + FULV (black arrows, apoptotic cardiac muscle fibers; blue arrow, markedly dilated congested blood vessel; yellow arrow, perivascular edema); **G** sham + entacapone (black arrow, scattered apoptotic cardiac muscle fibers; yellow arrow, markedly dilated congested blood vessels; blue arrow, intact irregular pericardium; green arrow, pyknotic nuclei and bright eosinophilic cytoplasm); **H** OVX + DOX + 2ME (green arrow, scattered apoptotic cardiac muscle fibers; black arrow, mildly congested blood vessel; blue arrow, irregular intact pericardium); **I** OVX + DOX + E2 (blue arrow, mild apoptotic cardiac muscle fibers; green arrow, average cardiac muscle fibers; black arrow, mildly congested intervening blood capillaries; yellow arrow, average blood vessel)

Subsequently, to evaluate the changes in cardiac tissues following DOX and/or ER agonist or antagonist treatments, the histopathological staining by H&E stain and examination of cardiac tissues were carried out. Control sham and control OVX heart displayed average pericardium, viable cardiac muscle fibers with distinct cell borders and central oval/elongated nuclei, and average blood vessels (Fig. 3A, B). The DOX treatment resulted in scattered apoptotic cardiac muscle fibers, mildly dilated blood vessels with mild interstitial edema, and extravasated red cells (Fig. 3C). When DOX was combined with surgical OVX, this led to marked apoptotic cardiac muscle fibers, moderately dilated blood vessels, mildly congested blood capillaries, and mild perivascular edema (Fig. 3D). These effects increased significantly when DOX was combined with chemical OVX by FULV (Fig. 3E) or combined with both chemical and surgical OVX (Fig. 3F). The histopathological alterations of cardiac tissues were decreased when the DOX was combined with ER agonist and 2ME (Fig. 3H, I).

**Fig. 4:**
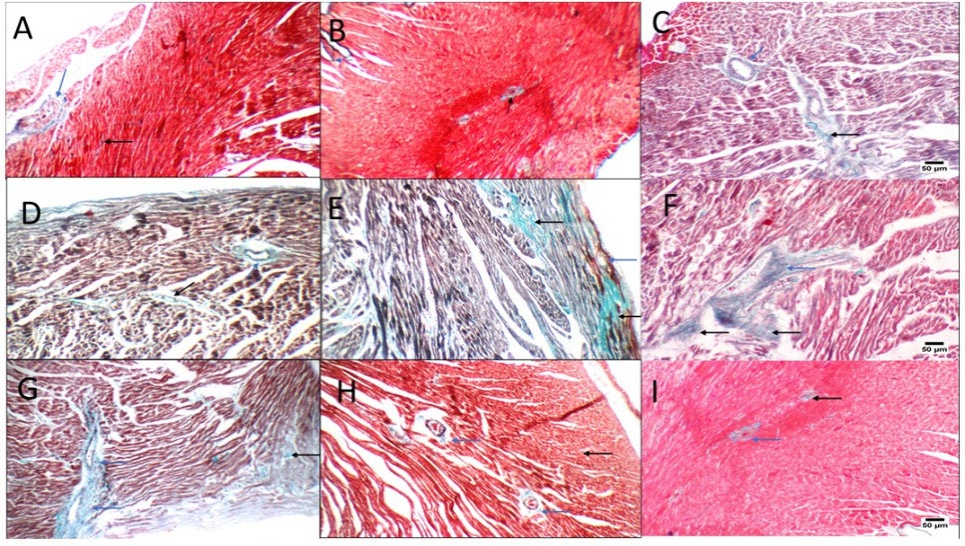
MTC staining of cardiac tissues following different treatments. **A** Control sham (black arrow, average collagen distribution in the myocardium; blue arrow, average collagen distribution around blood vessels); **B** control OVX (black arrow, average collagen distribution in the myocardium; blue arrow, average collagen distribution around blood vessels); **C** sham + DOX (black arrow, excess collagen fibers in the myocardium; blue arrow, average collagen distribution around blood vessels); **D** OVX + DOX (black arrow, excess collagen fibers in the myocardium; blue arrow, excess collagen fibers around blood vessels); **E** sham + DOX + FULV (black arrow, cardiac wall showing excess collagen in the myocardium); **F** OVX + DOX + FULV (black arrow, excess collagen fibers in the myocardium; blue arrow, excess collagen fibers around blood vessels); **G** sham + entacapone showing moderate fibroplasia; H OVX + DOX + 2ME (black arrow, average collagen distribution in the myocardium; blue arrow, average collagen distribution around blood vessels); I OVX + DOX + E2 (black arrow, average collagen distribution in the myocardium; blue arrow, moderate collagen fibers in the myocardium)

**Fig. 5:**
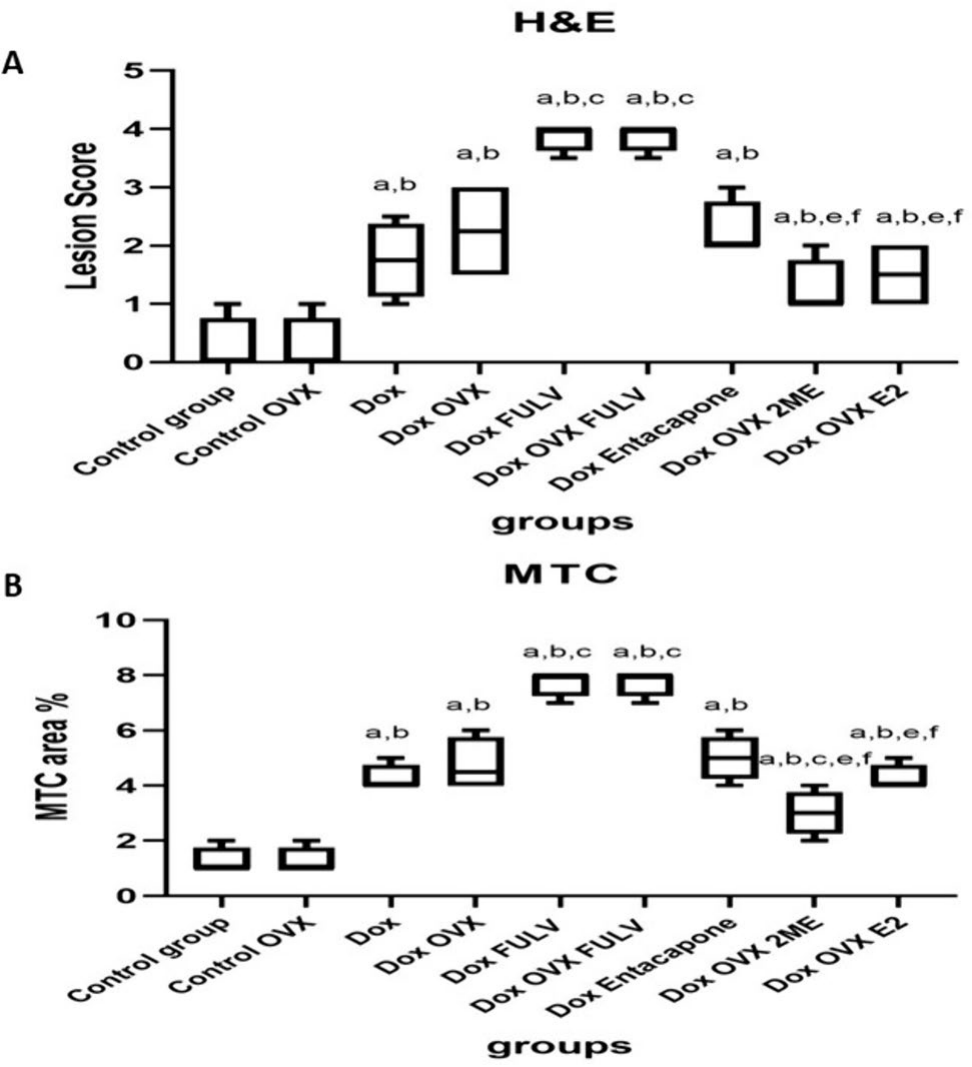
Statistical analysis of H&E lesion score (**A**) and MTC area % (**B**) of stained sections following different treatments. a, significantly different from control group A; b, significance from control OVX group B; c, significance from DOX-treated group C; d, significance from DOX + OVX group D; e, significance from DOX + FULV group E; f, significance from DOX + OVX + FULV group F; and g, significance from DOX + entacapone group G. Data represent mean ± SD. Multiple comparisons analyzed by ANOVA followed by Tukey-Kramer as a post hoc test (n = 8), p ≤ 0.05

Similarly, examination of MTC-stained sections of the control group and OVX control revealed the normal structure of cardiac muscles (Fig. 4A, B) with normal fine strands of fibrous tissue around blood vessels. Meanwhile, abundant fibrosis was noticed in the animals treated with DOX in addition to FULV or DOX with OVX and FULV groups (Fig. 4E, F). Moderate fibroplasia was noticed in stained sections from DOX, OVX+DOX, and entacapone groups (Fig. 4C, D, G). Mild cardiac myofibers were detected in 2ME and E2 groups (Fig. 4H, I). Likewise, statistical analysis of H&E lesion score (Fig. 5A) and area % of MTC (Fig. 5B) of stained sections showed that DOX induced significant changes in cardiac tissues compared to control groups, and this effect was exacerbated by reducing E2 in OVX and FULV groups and improved by administration of E2 and 2ME.

### Effect of different treatments on lipid-related metabolites

**Fig. 6:**
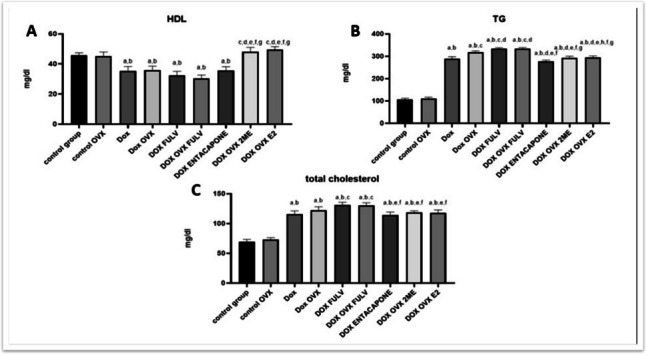
Effect of different treatments on lipid-related metabolites. A HDL; B TG; and C total cholesterol. a, significance from control group **A**; b, significance from control OVX group **B**; c, significance from DOX-treated group **C**; d, significance from DOX+OVX group **D**; e, significance from DOX+FULV group **E**; f, significance from DOX+OVX+FULV group **F**; and g, significance from DOX + entacapone group **G**. Data represent mean ± SD. Multiple comparisons were analyzed by ANOVA followed by Tukey-Kramer as a post hoc test (n = 8), p ≤ 0.05

In order to examine the consequences of different treatments on the serum level of the metabolic end products, the lipid components total cholesterol, HDL, and TG were quantified (Fig. 6). Data showed that the control groups did not significantly alter lipid profile parameters. DOX treatment on sham animals or DOX+ OVX increased serum levels of both TG and total cholesterol and decreased the level of HDL. This effect was more obvious in animal groups treated with ER blocker with DOX or DOX + OVX. Besides, entacapone increased serum total cholesterol and TG and decreased HDL but with a lower degree than FULV-treated groups. On the other hand, the treatment with ER agonist E2 and its metabolite 2ME maintained HDL at a higher level than other groups and decreased the serum levels of both TG and total cholesterol than DOX, DOX + OVX, DOX + FULV, and DOX + OVX + FULV groups.

### E2 and 2ME guard against DOX-induced cardiotoxicity through reserving the expression of CPT-1B and GLUT4

**Fig. 7:**
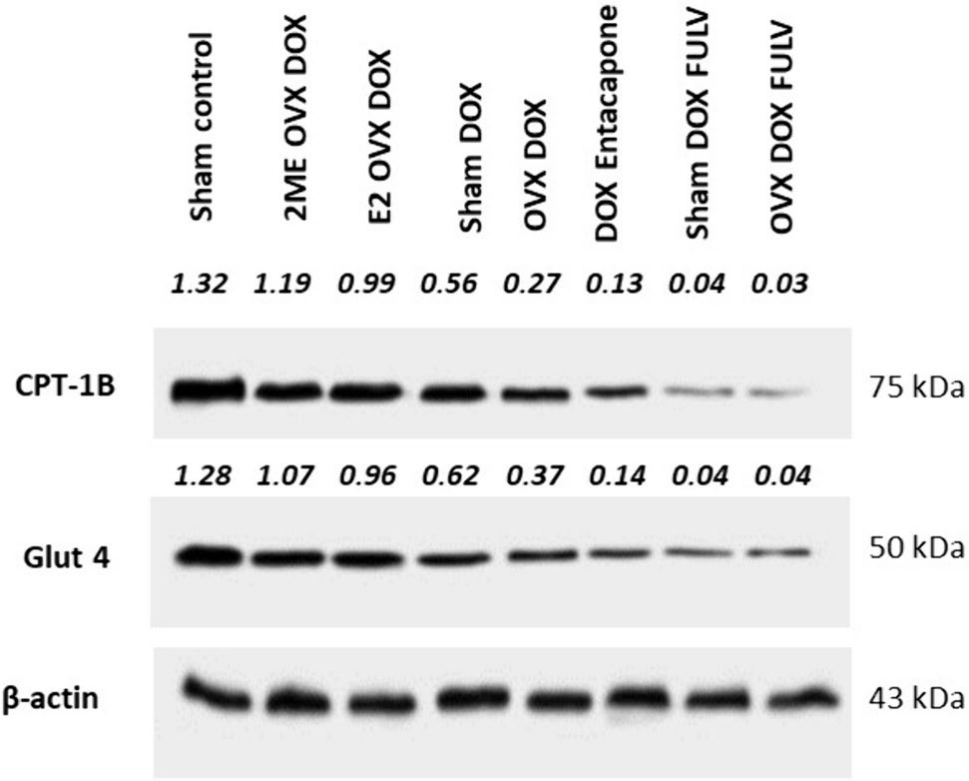
Effect of different treatments on metabolic enzymes CPT-1B and GLUT4 in cardiac tissues by western blot analysis

The expression levels of the key metabolic enzymes GLUT4 and CPT-1B were investigated by western blotting (Fig. 7). Treatment with DOX decreased the levels of both CPT-1B and GLUT4. The pronounced abolishment of CPT-1B and GLUT4 levels was shown in OVX and FULV groups. Entacapone had the same effect as OVX and FULV, but with FULV, the effect was more obvious. In contrast, 2ME and E2 treatment seemed to preserve GLUT4 and CPT-1B levels.

## Discussion

DOX cardiotoxicity is a serious problem that has restricted its use (Al-Malky et al 2020; Al-Shabanah et al 2012). The ways by which DOX affects cardiomyocytes have been elusive, and despite wide investigation, the precise mechanisms remain obscure. The oxidative stress made by DOX was thought of as the chief cause of heart damage; however, other processes are being explored aiming to improve cardioprotective strategies (Dallons et al 2020). Metabolic reprogramming has been recently investigated as a mechanism, which may have an impact on DOX-induced cardiotoxicity. For example, disruption of mitochondrial respiration and fatty acid oxidation in DOX-exposed cardiomyocytes resulted in cardiotoxicity in mice (Chen Ni et al 2019). Moreover, recent metabolomic studies indicate that persistent activation of glycolysis and impairment of oxidative phosphorylation, which are usually perceived after DOX treatment, may weaken the capacity of cardiac cells to meet the required energy, ultimately causing an energetic drop (Russo et al. 2021). Thereby, compounds regulating metabolic reprogramming have been recently inspected as protecting agents against DOX cardiotoxicity (Liu et al 2021). Several data point to that E2 can suppress DOX-induced myocardial damage through various mechanisms (Pokrzywinski et al 2018; Rattanasopa et al 2019) and 2ME has also been under investigation for its protective role on the heart (Docherty et al 2019; Maayah et al 2018). Consequently, in the current study, we investigated the effect of the E2 metabolite, 2ME on DOX-induced cardiotoxicity and the impact of metabolic reprogramming on this effect.

Cardiac remodeling is a group of molecular, cellular, and interstitial alterations that reveal clinical transformations in the heart shape and functions after injury (Azevedo et al 2016). Exposing the heart to radiation or anticancer drugs such as DOX can induce cardiac remodeling by the initiation of inflammation, fibrosis, vascular remodeling, hypertrophy, and others (Panpan et al 2022). In the current research, we were interested in studying whether E2/2ME2 would mitigate cardiac remodeling induced by DOX. Therefore, we evaluated several parameters, e.g., ECG, histopathology, and the cardiac-related enzymes CK-MB and LDH as well as the lipid profile markers TC, TG, and HDL. Together with former studies (Villani et al 1986; Wu et al 2016), our data indicated that DOX treatment on sham animals induced ECG changes in the heart manifested by both QTc and QT prolongation with an increase in T wave amplitude and ST segment elevation. This was accompanied by a rise in cardiac enzymes CK-MB and LDH levels. Elevation of these two enzymes signifies their leak from the injured cardiomyocyte membranes into the circulatory system and indicates cardiotoxicity (Zilinyi et al 2018). Histopathologically, DOX treatment resulted in changes in the heart muscles manifested as apoptotic cardiac muscle fibers, dilated blood vessels, interstitial edema, and extravasated red cells. These changes were more markedly observed when DOX was combined with surgical OVX and increased significantly when combined with chemical OVX by FULV or combined with both chemical and surgical OVX. Interestingly, ECG changes, serum levels of both CK-MB and LDH, and histopathological alterations of cardiac tissues decreased when the DOX treatment was combined with ER agonist and 2ME. This confirms the protecting impact of E2 and 2ME on the DOX-induced cardiac damage. This was further endorsed by staining with MTC where decreasing the level of E2 and/or its metabolite 2ME by OVX, FULV, or entacapone displayed higher fibrotic lesions. This effect was reversed by treatment with 2ME. Indeed, previous reports indicated that ERα is protective against fibrosis in response to pressure overload in female rats (Cheng et al 2020). Moreover, the antifibrotic influence of 2ME is well documented in a previous study (Elzayat et al 2020). Also, it was previously proposed that the valuable effects of 2ME as an antifibrotic agent in cardiac tissue not only are due to its effect on high pressure and remodeling in pulmonary arteries but may be partly due to its direct anti-remodeling properties in the right ventricle (Tofovic et al 2009). For more examination as to the consequences of different treatments on the serum level of the metabolic end products, the lipid components total cholesterol, HDL, and TG were quantified. It was noticed that DOX significantly reduced HDL and increased TG and total cholesterol levels. Reducing E2 levels by OVX and/or FULV raised total cholesterol and TG induced by DOX. On the other hand, exogenous E2 and 2ME restored HDL levels to normal but did not reverse the increase in TG and total cholesterol induced by DOX. It is noteworthy that E2 and 2ME have long been recognized for being protective against coronary heart disease by lowering cholesterol levels (Liu and Bachmann 1998). Also, a recent study concluded that lack of E2 in dogs can impair their lipid profiles (TG and VLDL levels were increased, while HDL was significantly decreased but not cardiac performance impairment 1 year following ovariohysterectomy) (Boonyapakorn et al 2020). Similarly, E2 and the polyphenolic compound silibinin which can modulate E2 receptor activation both improved the lipid profile and heart risk biomarkers in ovariectomized rats (Maleki et al 2022). Likewise, serum 2ME was positively associated with HDL in another study (Masi et al 2012) and managed to reduce atherosclerosis in vivo (Bourghardt et al 2007).

A prominent body of data supports the notion that impaired metabolic reprogramming contributes to DOX cardiotoxicity (Ni et al 2019). GLUT and CPT families are important key players in glucose and lipid metabolism. Clinical and animal studies have presented varied results as to the role of CPT-1 on the heart (Yu et al 2022a, b). For example, specific CPT-1 inhibitors exerted protective effects against heart failure and cardiac hypertrophy (Schmidt-Schweda and Holubarsch 2000), but failed to reverse induced heart failure due to pressure overload in vivo (Schwarzer et al 2009). Moreover, CPT-1B deficiency triggered lipotoxicity in the cardiac cells under stress, causing worsening of cardiac pathology (He et al 2012) and inducing cardiac hypertrophy as well as mortality in mice (Haynie et al 2014). Thus, at the molecular level, to explore the mechanisms by which E2 and 2ME guard against the DOX-induced cardiotoxicity, the expression levels of GLUT4 and CPT-1B were investigated. Our data indicated that DOX decreased the levels of both CPT-1B and GLUT4. The abolishment of their levels was shown in OVX and FULV treatment. Entacapone had the same effect as OVX/FULV treatment, but with FULV, the effect was more pronounced. In contrast, 2ME and E2 preserved GLUT4 and CPT-1B levels. This observation is supported by data from the literature. For example, GLUT4 protein was downregulated in the adipose tissues of DOX-treated rats (Biondo et al 2016). Also, E2 treatment promoted GLUT4 translocation from the plasma membrane, therefore improving insulin sensitivity and glucose uptake in 3T3-L1 adipocytes (Campello et al 2017). Additionally, it was suggested that an impaired circulating imbalance of the ESR1/ESR2 ratio might have significant concerns in metabolism (Gregorio et al 2021). It is also reported that DOX inhibits the CPT system and the subsequent transport of long-chain fatty acids across the mitochondrial membranes, and that a main cause of DOX cardiomyopathy is due to compromised fatty acid oxidation (Yoon et al 2003). On the other hand, it was suggested that E2-like compounds have a role in lipid metabolism by boosting important enzymes in the oxidative pathway of fat in skeletal muscles (Campbell and Febbraio 2001). For example, the phytoestrogen genistein was associated with improved activity of CPT and the rate of β-oxidation in the fat tissue of rats (Choi et al 2012).

It is noteworthy that the effects of exogenous E2 and exogenous 2ME were almost equivalent in most of the measured parameters. This suggests that the effect of E2 is partly due to COMT-mediated conversion of E2 to 2ME. It also implies that 2ME may be a beneficial replacement to E2 in protecting against DOX-induced cardiotoxicity. This is crucially important, especially in breast cancer patients where E2 can be involved in carcinogenesis. 2ME, however, is a safe alternative in these cases because it is devoid of any estrogenic effect. We also found out that when the E2 receptor blocker FULV was administered in some groups, this led to deterioration of DOX cardiotoxicity. This may be either due to preventing the protective effect of E2 or via the E2-independent estrogen receptor effect. Further studies are required to elucidate this point.

Accordingly, based on our data supported by literature, we document that E2 and its metabolite 2ME provide protection against DOX-induced cardiotoxicity at least in part via regulating the expression of metabolic enzymes GLUT4 and CPT-1B.

## Conclusion

The current study has verified that 2ME alleviated DOX-induced cardiotoxicity by regulating various biological processes. It modulated DOX-induced cardiac remodeling, e.g., ECG changes, cardiac-related enzymes (CK-MB and LDH), histopathological alterations, and lipid profile markers (total cholesterol, HDL, and TG). Importantly, its cardioprotective action was, at least in part, through metabolic reprogramming by regulating the expression of GLUT4 and CPT-1B proteins which are involved in glucose transport and fatty acid oxidation respectively. Our observations also shed the light that the cardioprotective action of E2 is partly due to its metabolite 2ME which is devoid of any estrogenic activity and fits both sexes. Hence, it is expected that it can be used safely in cancer patients of hormone-dependent types. Moreover, metabolic reprogramming modulators may have potential protective effects against DOX-induced cardiomyopathy.

## Supplementary Information

Below is the link to the electronic supplementary material.

Supplementary file1 (PDF 51 KB)

## Data Availability

Dataset generated from this study is available from the corresponding author upon request.
