# Supplementary figures and images for: 2-Methoxyestradiol ameliorates doxorubicin-induced cardiotoxicity by regulating the expression of GLUT4 and CPT-1B in female rats
Source: Naunyn Schmiedebergs Arch Pharmacol. 2024 Apr 23;397(9):7129–39. doi: 10.1007/s00210-024-03073-z (PMC11422279; doi:10.1007/s00210-024-03073-z)

## Uncropped Western blot

### CPT-1B

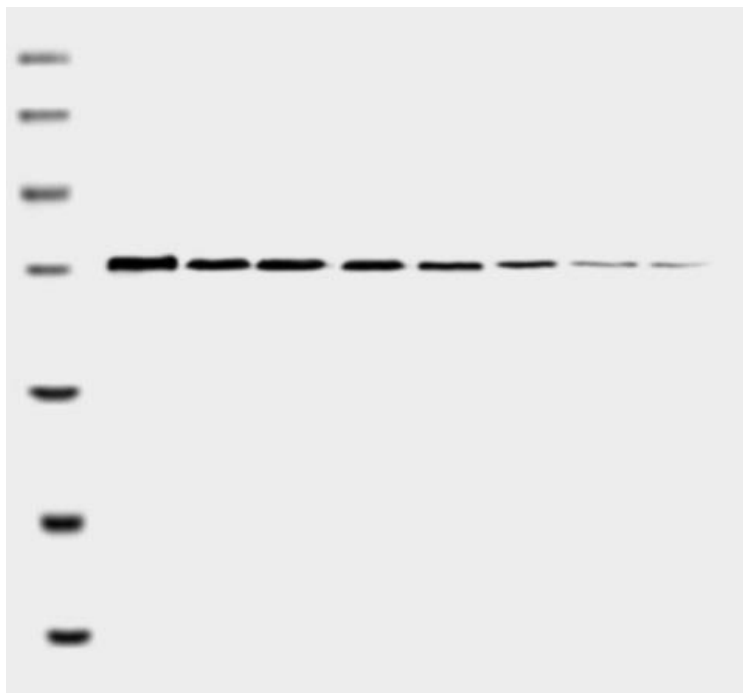

### GLUT4

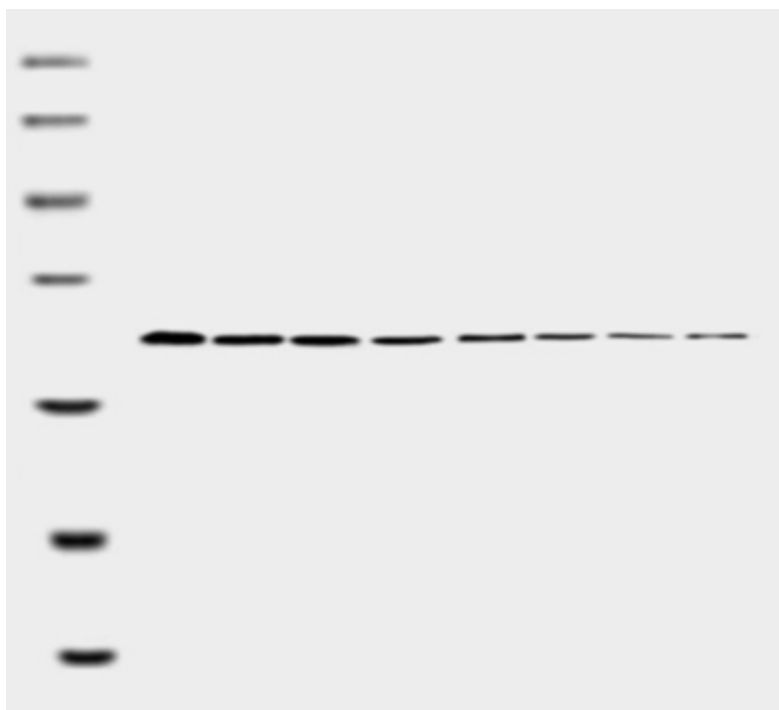

**B-actin**

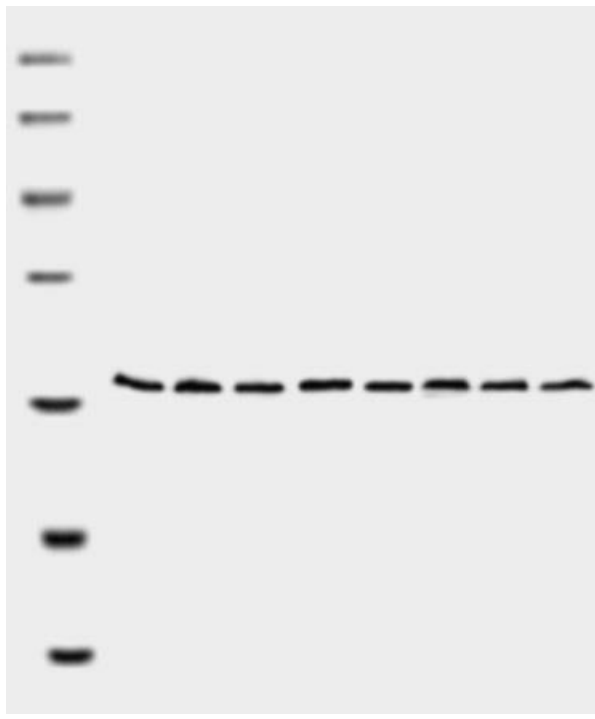

Supplement: Supplementary file 1 — Supplementary file1 (PDF 51 KB) [file 210_2024_3073_MOESM1_ESM.pdf]
